# Eccentric Muscle Contractions: Risks and Benefits

**DOI:** 10.3389/fphys.2019.00536

**Published:** 2019-05-03

**Authors:** Stéphanie Hody, Jean-Louis Croisier, Thierry Bury, Bernard Rogister, Pierre Leprince

**Affiliations:** ^1^Department of Motricity Sciences, University of Liège, Liege, Belgium; ^2^GIGA-Neurosciences, University of Liège, Liege, Belgium; ^3^Department of Neurology, The University Hospital Center, University of Liège, Liege, Belgium; ^4^GIGA – Laboratory of Nervous System Disorders and Therapy, University of Liège, Liege, Belgium

**Keywords:** skeletal muscle, eccentric contraction, exercise-induced muscle damage (EIMD), delayed-onset muscle soreness (DOMS), eccentric muscle training

## Abstract

Eccentric contractions, characterized by the lengthening of the muscle-tendon complex, present several unique features compared with other types of contractions, which may lead to unique adaptations. Due to its specific physiological and mechanical properties, there is an increasing interest in employing eccentric muscle work for rehabilitation and clinical purposes. However, unaccustomed eccentric exercise is known to cause muscle damage and delayed pain, commonly defined as “Delayed-Onset Muscular Soreness” (DOMS). To date, the most useful preventive strategy to avoid these adverse effects consists of repeating sessions involving submaximal eccentric contractions whose intensity is progressively increased over the training. Despite an increased number of investigations focusing on the eccentric contraction, a significant gap still remains in our understanding of the cellular and molecular mechanisms underlying the initial damage response and subsequent adaptations to eccentric exercise. Yet, unraveling the molecular basis of exercise-related muscle damage and soreness might help uncover the mechanistic basis of pathological conditions as myalgia or neuromuscular diseases. In addition, a better insight into the mechanisms governing eccentric training adaptations should provide invaluable information for designing therapeutic interventions and identifying potential therapeutic targets.

## Introduction

An eccentric (lengthening) muscle contraction occurs when a force applied to the muscle exceeds the momentary force produced by the muscle itself, resulting in the forced lengthening of the muscle-tendon system while contracting (Lindstedt et al., 2001). During this process, the muscle absorbs energy developed by an external load, explaining why eccentric action is also called “*negative work*” as opposed to concentric (shortening) contraction or “*positive work*” (Abbott et al., 1952). Although not always obvious, eccentric muscle contractions are an integral part of most movements during daily or sport activities. Skeletal muscles contract eccentrically to support the weight of the body against gravity and to absorb shock or to store elastic recoil energy in preparation for concentric (or accelerating) contractions (LaStayo et al., 2003b). The slowing-down role of suchcontractions is classically illustrated by downhill running or walking down the stairs during which the eccentric work of the knee extensor muscles is accentuated (Gault and Willems, 2013).

Compared to concentric or isometric (constant length) contractions, eccentric muscle actions possess several unique features that may be responsible for unique adaptations (Guilhem et al., 2010; Duchateau and Baudry, 2014). Greater forces are generated during eccentric contraction compared to other contraction types for a given angular velocity (Hortobagyi and Katch, 1990). In addition, eccentric contractions require less motor unit activation and consume less oxygen and energy for a given muscle force than concentric contractions (Abbott et al., 1952). Indeed, the metabolic cost required for eccentric exercise is approximately fourfold lower than for the same exercise performed concentrically. Reduced cardiorespiratory and hemodynamic responses have been reported following eccentric exercise when compared to concentric exercise at the same absolute workload (Overend et al., 2000; Meyer et al., 2003). While many questions remains unanswered, it is well accepted that neural strategies controlling eccentric contractions considerably differ from concentric or isometric contractions (Duchateau and Baudry, 2014). Differences are detected on the level of the contracting muscle as well as on the cortical level. Most studies indicate a reduced central activation (evidenced by a lower EMG amplitude) during maximal eccentric contractions than maximal concentric or isometric contractions. This has implications on eccentric coordination: fine motor control in eccentrically biased actions appears more difficult as fewer motor-units are required for the same work (Hoppeler, 2016). The twitch interpolation technique also revealed a greater voluntary deficit in eccentric compared to concentric contractions, such that untrained individuals are usually unable to fully activate their muscles during maximal eccentric muscle contractions. Further characteristics of eccentric contraction are a greater cortical excitability but a lower motor units discharge. Collectively, the mechanisms underpinning the unique features of eccentric contraction are not well understood (Hoppeler and Herzog, 2014).

Due to its specific physiological and mechanical properties, the eccentric contraction has gained a growing interest in several fields. Besides its interest in sport training or in physical medicine and rehabilitation (Croisier et al., 2002; Kjaer and Heinemeier, 2014; Vogt and Hoppeler, 2014), evidence is accumulating regarding the benefits of eccentric exercise in special populations of aged individuals or patients with chronic health diseases such as neuromuscular pathologies (Roig et al., 2008; Gault and Willems, 2013; Isner-Horobeti et al., 2013; Hyldahl and Hubal, 2014). Indeed, the two main defining properties of eccentric contraction “highest forces and lower energy requirement” makes this contraction regime a judicious alternative to conventional muscle training. To date, it is well accepted that the benefits of eccentric exercise transcend improved muscle function, as this mode of training has been shown to induce a number of favorable repercussions on neural drive or health-related factors (Paschalis et al., 2010, 2013). For many years, eccentric regime has been largely used in sport training to improve maximal muscular strength, power as well as coordination during eccentric tasks. Robust evidence support its wide prescription in the sport rehabilitation field, notably in the treatment of tendinopathies (Croisier et al., 2007; Kaux et al., 2013). In addition, implementing eccentric exercise in athletes showed its effectiveness to prevent sport injuries such as hamstring strain (Croisier et al., 2002). While research has mainly focused on the functional outcomes following eccentric resistance training using high-loads, the potential of low/moderate load regimes received much attention over the last decade (LaStayo et al., 2014; Hoppeler, 2016). With the increasing consideration of the physical activity in numerous medical fields over the last decades, a novel training modality based on low to moderate load ECC exercise has emerged. This modality, referred as RENEW (Resistance Exercise via Eccentric Work) by LaStayo et al. (2014), appears to result in similar gains in muscle strength and volume as traditional strength training. Since eccentric modality provides a strong mechanical stress at a lower metabolic cost (Lastayo et al., 1999), it appears particularly suitable for training individuals with medical conditions associated to muscle wasting and reduction in muscle strength, mobility and aerobic capacity (Hoppeler, 2016). Eccentric training is increasingly proposed to patients with cardiorespiratory problems, sarcopenia of old age, cachexia, diabetes type 2, neurological and musculoskeletal diseases (Julian et al., 2018). Along with the positive effects on the muscle function, aerobic eccentric exercise induces specific effects on muscle energetic metabolism, insulin resistance and blood lipid profile, reducing disease risks. It is thus recognized as a promising lifestyle factor to combat obesity and dyslipidemias (Paschalis et al., 2010; Julian et al., 2018, 2019).

However, despite the above-mentioned advantages, the use of eccentric exercise in clinical conditions has been frequently the object of contrasting opinions, because of its potential undesirable associated effects. Indeed, eccentric exercise induces greater muscle damage and negative functional consequences in an healthy naïve muscle than other types of exercise (Friden and Lieber, 1992). Indeed, the combination of high force and reduced recruitment of fiber number during eccentric contractions causes a high mechanical stress on the involved structures that may lead to focal microlesions of the muscle fibers (Lieber and Friden, 1999). Numerous histological studies described widespread Z-line streaming with myofibrillar disruption and necrosis following intense and/or unaccustomed eccentric exercise (Friden and Lieber, 1998; Crameri et al., 2007; Lauritzen et al., 2009). The sarcomeric disorganization has been associated with disruptions to the sarcolemma and the extracellular matrix, swelling of mitochondria, dilation of the transverse tubule system and fragmentation of the sarcoplasmic reticulum (Takekura et al., 2001; Crameri et al., 2004). Sarcolemmal disruption may be highlighted by the appearance of sarcoplasmic proteins into the blood (as creatine kinase, CK and myoglobin, Mb) or by the cytoplasmic accumulation of proteins that are normally not present in muscle fibers (as albumin and immunoglobulins) (McNeil and Khakee, 1992; Clarkson and Hubal, 2002). Vital dye such as Evans blue is also used in rodents to demonstrate increased sarcolemmal permeability (Hamer et al., 2002). Damage to extracellular matrix and connective tissue components also occur following a novel eccentric exercise (Brown et al., 1997; Crameri et al., 2007). Morphological abnormalities observed immediately after exercise gradually extent to a larger number of muscle fibers and appear exacerbated 2–3 days post-exercise (Friden et al., 1983a). These observations have led authors to define primary and secondary damage phases (Morgan and Allen, 1999). Both human and animal studies supported that Type II (in particular IIb) muscle fibers are more damaged after eccentric exercise than Type I fibers (Friden et al., 1983b; Jones et al., 1986; Lieber and Friden, 1988). Several hypotheses could explain the higher susceptibility of Type II fibers to exercise-induced muscle damage (EIMD). Among these are differences in their structural composition (Z-line, fiber type specific protein isoforms such as titin), a reduced oxidative capacity, a lower ability to regulate calcium homeostasis or a selective recruitment of fast-twitch muscle fibers during eccentric contraction (Lieber and Friden, 1999; McHugh et al., 1999a; Byrne et al., 2004).

The EIMD manifests itself by a range of clinical symptoms including delayed-onset muscle soreness (DOMS), stiffness, swelling and various functional deficits such as a loss in force generating capacity or decreased proprioceptive function (Clarkson, 1992). To avoid the invasive nature of muscle biopsies, these clinical manifestations as well as the plasma CK activity are frequently used to indirectly assess the presence of muscle damage (Warren et al., 1999; Clarkson and Hubal, 2002). The magnitude of changes in EIMD indirect markers (in particular, the plasma CK activity) shows a marked inter-individual variability even when subjects are submitted to standardized eccentric protocols (Clarkson et al., 1992; Nosaka and Clarkson, 1996; Hody et al., 2013c). Multiple factors as muscle architecture, muscle typology, individual fitness, age, sex, and genetic variability may contribute to the wide inter-subject variability in the response to eccentric exercise (Vincent and Vincent, 1997; Clarkson and Hubal, 2002; Yamin et al., 2007; Hody et al., 2009; Hyldahl and Hubal, 2014). Even if DOMS and associated clinical symptoms spontaneously disappear after few days, these negative consequences can delay or disturb rehabilitation and/or training programs. Exercise is necessary to maintain a good health and to prevent physical inactivity-related diseases, but unpleasant sensations resulting from unaccustomed exercise can discourage people to continue physical activity. Moreover, due to the mechanical fragility, the risk of further injuries (e.g., muscle tears or ligament rupture) increases if intense physical activities are performed during a DOMS episode or the following days (Nicol et al., 2006). It is worth noting that muscle soreness disappears before the full recovery of muscle function, further elevating the injury incidence (Strojnik et al., 2001). Although exceptional, extreme CK and Mb elevations associated with EIMD could be severe enough to provoke a kidney tubulopathy (Sayers et al., 1999).

Given the risks and drawbacks related to the occurrence of EIMD described above, the development of strategies to prevent or reduce the intensity of its clinical manifestations has become a primary goal of many studies. The most commonly used approaches include stretching, cryotherapy, electric or manual therapies, whole-body vibration or nutritional and pharmacological interventions (Cheung et al., 2003; Barnett, 2006; Bloomer, 2007; Howatson and van Someren, 2008). Despite the large number of clinical trials, there are very few evidence-based guidelines for the application of these interventions. The inconsistencies in the dose and frequency of the investigated interventions may account for the lack of consensus regarding their efficacy. Conversely, there is unequivocal evidence that a first bout of eccentric exercise confers protection against EIMD following a subsequent bout of the similar exercise. This muscle adaptation process, commonly called “*the repeated-bout effect*” (RBE), is characterized by reduced increases in muscular proteins in the blood, attenuated DOMS, less muscle swelling, reduced abnormality in echo intensity of B-mode ultrasound and/or magnetic resonance images and faster recovery of muscle strength and range of motion following the repeated bout (McHugh, 2003; Nosaka and Aoki, 2011). Although a significant protective effect occurs after a single eccentric bout (Clarkson et al., 1992; Nosaka and Clarkson, 1995), the adaptive process appears more complete after several sessions (Croisier et al., 1999; Hody et al., 2011). The RBE seems to imply long-lasting adaptation since it persists for several weeks and even up to 6 months but the magnitude of the protection decreases over time (Nosaka et al., 2001, 2005). It is interesting to note that the magnitude of the protective effect is not necessarily dependent on the severity of the initial muscle damage. It has been demonstrated that repeating bouts of “non-damaging” eccentric exercise can provide strong protective adaptations against subsequent bouts of maximal eccentric exercise (Chen et al., 2013). Therefore, to date, performing repeated sessions with submaximal eccentric contractions appears to be the most efficient strategy to induce eccentric training-induced adaptations that would prevent further EIMD and DOMS. The demonstration that eccentric actions can be performed without damage and soreness allowed considering the potential of eccentric trainings in medical conditions. Studies conducted first with healthy subjects and then, with patient populations, have supported the application of eccentric trainings as a safe, feasible and efficient strategy for rehabilitation purposes (LaStayo et al., 2000; Hoppeler, 2016). Numerous studies have attempted to elucidate the mechanisms underlying the RBE, but this feature of the skeletal muscle is not fully understood (McHugh et al., 1999a; McHugh, 2003; Nosaka and Aoki, 2011).

Despite considerable amount of available data at the clinical and histological levels, a significant gap still remains in the understanding of the mechanisms that mediate morphological, cellular, and molecular responses to muscle damaging eccentric exercise (Hoppeler and Herzog, 2014). In addition, the molecular events underlying the specific eccentric training muscle adaptations are not fully understood (McHugh, 2003; Nosaka and Aoki, 2011). This review begins by describing the potential mechanisms leading to muscle damage and soreness following unaccustomed eccentric exercise. Then, are discussed the current knowledge of the eccentric training-induced adaptations including the main hypotheses of the protective effect against EIMD. Finally, the multiple applications of eccentric training, justifying the need of an improved understanding of its underlying molecular and cellular mechanisms, are exposed.

## Unaccustomed Eccentric Exercise

### Mechanisms of Exercise-Induced Muscle Damage

It is generally accepted that the damage process is initiated due to a lack of homogeneity in sarcomeres stretching (asymmetric lengthening). This theory initially proposed by Morgan (1990) suggests that during eccentric contractions, the weakest sarcomeres or even half-sarcomeres will absorb most of the length change (Morgan, 1990). These may be stretched beyond the point of myofilament overlap resulting in disrupted or “popped” sarcomeres. In line with this proposal, several studies have clearly shown that the length of the muscle during eccentric contraction is a critical factor in determining the extent of damage (Talbot and Morgan, 1998). Eccentric contractions performed at longer muscle length results in greater symptoms of damage than similar contractions at shorter muscle length (Lieber and Friden, 1993).

**FIGURE 1:**
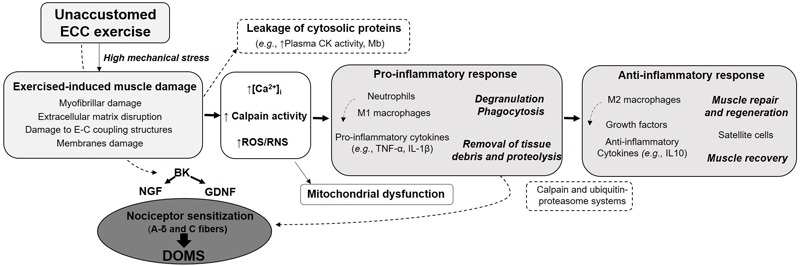
Summary of the main specific features of eccentric contraction, its multi-target beneficial effects and potential risks associated with unaccustomed and/or maximal eccentric exercise.

The initial mechanical damage would trigger a cascade of events leading to more severe secondary damage (Figure 1). Loss of calcium homeostasis, possible inflammatory reaction and reactive oxygen species (ROS) production are thought to contribute to the secondary damage phase. The disturbances in Ca^2+^ homeostasis observed following unaccustomed eccentric exercise may be the consequence of membrane damage (Friden and Lieber, 2001) or opening of stretch-activated channels (Overgaard et al., 2002). Abnormal increase in calcium concentration inside muscle cells is responsible for the activation of muscle proteases, named calpains. Since these proteases cleave important structural proteins in charge of myofibril integrity (as desmin and alpha-actinin), they have been suggested to contribute to EIMD. The degradation of proteins released from myofibrillar structures by the calpains could be enhanced by other proteolytic pathways as the ubiquitin–proteasome system (Raastad et al., 2010). Activation of calpains may also result in the destruction of membrane constituents, which in turn, will increase calcium entry. Elevated calcium concentrations in skeletal muscle mitochondria, which can alter mitochondrial respiratory function, also occur following unaccustomed eccentric exercise (Rattray et al., 2011, 2013). This calcium overload may be associated with the opening of the mitochondrial permeability transition pore (mPTP) leading to the activation of cell death signaling or with the increased calpain proteolytic activity which is capable of targeting proteins resulting in mitochondrial dysfunction. Furthermore, the increased calpains activity can promote neutrophils and macrophages activation, leading to ROS production (Powers and Jackson, 2008). Besides the clinical symptoms associated with EIMD (such as DOMS and decline in muscle strength), EIMD have been reported to induce metabolic consequences at the acute phase: decreased glucose uptake and insulin sensitivity, impaired glycogen synthesis, elevated metabolic rate and a shift toward non-oxidative metabolism (Tee et al., 2007).

### Inflammatory and Immune Responses to Eccentric Exercise

While the development of an inflammatory reaction after eccentric exercise has been debated (Yu et al., 2002; Malm and Yu, 2012), many studies have now provided clear evidence of systemic and local inflammatory responses in both rodents and humans following various types of eccentric exercise (Peake J. et al., 2005; Paulsen et al., 2012). However, in contrast to extensive works describing the histological and clinical signs associated to EIMD, the mechanisms underlying the inflammation-immune responses and the subsequent regenerative events are less well understood (Peake J. et al., 2005; Paulsen et al., 2012). The inflammation processes following damaging exercise was initially considered as a detrimental event due to its association with muscle damage, soreness and delayed recovery but it is now well accepted that the inflammatory stages are crucial for functional recovery of the muscle to EIMD. The inflammation would ensure the removal of tissue debris from the injured area and promote muscle repair by activating muscle cells. Over the last decade, more studies have focused on the implication of multiple immune cell types interacting with the muscle and emphasized the undeniable role of satellite cells for muscle regeneration following one bout of eccentric exercise (Crameri et al., 2004; Paulsen et al., 2012).

Early accumulation of leukocytes, primarily neutrophils, has been observed in micro-blood vessels of the damaged muscle, as well as in the perimysium, immediately after exercise. In case of moderate to severe EIMD, histological studies have consistently shown that neutrophils infiltrate into the muscle and accumulate in the damaged area from 1 and 24 h after eccentric exercise (Paulsen et al., 2010). It is likely that secretion and/or passive release of chemoattractant proteins due to modifications to membrane permeability are involved in the recruitment of circulating inflammatory cells. They initiate the pro-inflammatory stage through phagocytosis and by releasing proteolytic enzymes (such as elastase or myeloperoxidase) and reactive species. At later time points, when neutrophils are cleared from muscle, pro-inflammatory macrophages start to accumulate. This type of macrophages, referred as M1, contribute to the phagocytosis of the damaged tissue by secreting pro-inflammatory cytokines (e.g., TNF-α, IL-6, and IL-1β) and secretory leukocyte protease inhibitor. Tissue-resident monocytes may also become activated after exercise, in addition to the leukocytes originating from the blood circulation. Neutrophils and M1 macrophages interact with each other to regulate the proinflammatory response of muscle damage. Their influx inside injured myofibers appears to be dependent on the magnitude of EIMD and may lead to an exacerbation of the initial cellular alterations. Conversely, M2 macrophages that appear later generally produce anti-inflammatory cytokines and signaling molecules involved in the muscle recovery and regeneration. Large variations across healthy individuals are observed, some presenting substantial leukocyte accumulation whereas others displayed very little leukocytes invasion. Furthermore, the magnitude of the inflammation response appears to be dependent on the initial perturbations induced by the exercise. It is assumed that minor perturbations result in a cell-signaling-mediated adaptive response, whereas intense eccentric actions seem to generate a more severe response leading to secondary damage to myofibers and increased risk of necrosis. While significant necrosis is observed after electrically stimulated contractions, segmental myofiber necrosis may occur without affecting the whole myofiber, even in severe cases of EIMD. Interestingly, the degree of leucocyte accumulation seems to be related to the changes in force-generating capacity of the muscle (Paulsen et al., 2010). Therefore, measuring the decline of muscular strength following exercise, which is recognized as the best indirect marker of EIMD, may inform on the status of the muscle. In contrast, the level of leukocyte invasion into injured myofibers is not necessarily related to DOMS.

The muscle inflammatory response appears to intimately coregulate with muscle regeneration. Indeed, additionally to their immune functions, macrophages also participate to myogenesis and contribute to the extracellular matrix remodeling. M1 macrophages stimulate satellite cells proliferation whereas M2 macrophages interact with differentiating satellite cells (Paulsen et al., 2012). The latter can also promote general protein synthesis within muscle fibers. The replacement of macrophages M1 to anti-inflammatory M2 macrophages is a key stage for the transition from proinflammatory to anti-inflammatory stages. This process is regulated by different signals including the phagocytosis of cell debris, IL10 and AMP-activated protein kinase (Chazaud, 2016). While a large body of research has primarily focused on neutrophils and macrophages, other cell types interact with the muscle and are important in the inflammation and muscle regeneration processes. These include notably mast cells, T lymphocytes, eosinophils, fibro-adipogenic progenitors, and pericytes (Paulsen et al., 2012).

The inflammation and immune responses are mediated by various growth factors and the actions of exercise-responsive cytokines (i.e., IL-6, CCL2, and interferon-γ), pro-inflammatory cytokines (TNF-α and IL-1β) and the anti-inflammatory cytokine IL-10. Collectively, all these cytokines appear to activate myoblast proliferation and some of them are involved in myoblast differentiation (Peake J. et al., 2005). Interestingly, the satellite cells activity is differentially affected by the contraction mode in human muscle following exercise of the same work load. Resistance eccentric, but not concentric, exercise has been shown to elicit the proliferation of satellite cells immediately after exercise, suggesting that EIMD is the main stimulus for activating the satellite cells pool (Hyldahl and Hubal, 2014; Hyldahl et al., 2014).

The precise source of production for cytokines found in the circulation during and after exercise is not well established. Indeed, the cytokines can be produced not only by leucocytes, but also by myofibers and peri-tendinous tissue (Paulsen et al., 2010). The term “myokines” has been introduced to refer to muscle derived-cytokines and chemokines. Myokines are secreted by the skeletal muscle in order to communicate with non-muscle tissues and act as auto-, para- and endocrine mediators. These might be molecular mediators which link muscle exercise and the whole body physiology (Schnyder and Handschin, 2015). While research to date has focused primarily on the biological functions of the myokines in regulating metabolism, much less attention has been made regarding their role in inflammatory and adaptation to EIMD (Paulsen et al., 2010). Nevertheless, studies investigating the cytokine responses to eccentric exercise demonstrated increased activity of some cytokines such as MCP-1 and IL-10 after eccentric but not concentric exercise (Hyldahl and Hubal, 2014). The anti-inflammatory cytokine IL-10 may attract T lymphocytes, which activate muscle cell proliferation and muscle regeneration. Systemic increase of IL-8 and upregulation in intramuscular IL-8 mRNA expression and plasma levels after downhill running and eccentric actions of the quadriceps has also been reported (Hubal et al., 2008; Buford et al., 2009). IL-8 plasma levels are also increased after eccentric muscle contractions, but unchanged following concentric exercise. IL-8 is known to attract primary neutrophils but this chemokine may also promote neovascularization of muscle tissue through its association with CXCR2 (Schnyder and Handschin, 2015). Some studies also showed an increase in plasma concentration of IL-1ra and G-CSF (granulocyte-colony stimulating factor) in the hours after eccentric exercise (Peake J. et al., 2005; Peake J.M. et al., 2005).

A large body of science has focused on IL-6. This myokine, considered as one “exercise factor,” is regulated by exercise and acts both locally within the muscle and on distal organs in an endocrine-like fashion (Catoire and Kersten, 2015). IL-6 has initially been characterized as a prototypical pro-inflammatory cytokine by contributing to neutrophil mobilization and activation and promoting impaired peripheral insulin resistance. In contrast, anti-inflammatory properties of IL-6 have been proposed later. Indeed, its exercise-induced systemic increase generates the elevation of plasma level of several anti-inflammatory cytokines (IL-1ra and IL-10) and inhibits the production of the pro-inflammatory cytokine TNF-α (Pedersen and Febbraio, 2008; Pedersen, 2012). Various cell types secrete IL-6, including the skeletal muscle fibers during and after exercise. Alongside the systemic increase, IL-6 mRNA levels are augmented in contracting muscle fibers. IL-6 is considered as an energy sensor of the muscle (Pedersen, 2012) since its secretion from the exercising muscles increases glucose uptake and fatty acid oxidation locally and improves insulin secretion, which further increases glucose uptake into muscle fibers. Hepatic glucose delivery and fatty acid release from adipose tissue are also stimulated supporting the maintenance of metabolic homeostasis during exercise (Febbraio and Pedersen, 2002). Muscle derived IL-6 was first thought to be related to injury but “non-damaging exercise” has been shown to lead to substantial IL-6 increase (Croisier et al., 1999). Nevertheless, IL-6 contributes with TNF-α and MCP-1 to muscle regeneration after EIMD by stimulating the proliferation and differentiation of myoblasts (Schnyder and Handschin, 2015). Moreover, the transforming growth factor-beta is another cytokine involved in muscle recovery and repair after muscle damage that regulates extracellular matrix remodeling and promotes fibrosis (Kim and Lee, 2017).

### Delayed-Onset Muscle Soreness

Delayed-onset muscle soreness (DOMS) refers to unpleasant, dull, aching pain, usually felt during palpation, contraction or stretching of the affected muscle. Such muscle soreness typically appears 12–24 h after unaccustomed eccentric exercise, peaks at between 24 and 72 h before progressively subsiding and disappearing within 5–7 days post-exercise. Interestingly, DOMS intensity is poorly correlated with other EIMD indirect markers and seems thus not to reflect the magnitude of muscle damage (Nosaka et al., 2002). Although DOMS is an extremely common symptom, why DOMS occurs with a delay, and why eccentric contraction but not shortening contraction induces DOMS is not clearly understood. Several hypotheses have been put forward to explain the mechanism of DOMS. These include lactic acid release, spasm, connective tissue damage, muscle damage, inflammation and oxidative stress (Hyldahl and Hubal, 2014). For many years, the most widely supported hypothesis was that the biochemical, thermal and mechanical changes associated with the inflammatory response sensitize small diameter muscles afferents (types III and IV) that may then be at the origin of the sensation of muscle soreness (Friden and Lieber, 1992). It was only in 2010 that Murase and coworkers provided new insights into the molecular mechanisms of DOMS generation. They highlighted bradykinin and nerve growth factor (NGF) as important players in the development of DOMS following eccentric contractions (Figure 1). Using a rodent model, they demonstrated that a bradykinin-like substance released from the muscle during eccentric exercise triggers the process of muscular mechanical hyperalgesia by upregulating NGF through B2 receptors in exercised muscle of rats. In humans, NGF has been shown to be involved in the generation and potentiation of pain following eccentric exercise (Nie et al., 2009). Another pathway proposed to be involved in the development of DOMS is the activation of the COX-2-glial cell line-derived neurotrophic factor (GDNF) (Murase et al., 2013; Mizumura and Taguchi, 2016). Similarly to NGF pathway, this agent likely generates muscle mechanical hyperalgesia directly by stimulating muscle nociceptors, or by binding to extracellular receptors. While myofibers micro-damage were believed to be necessary to initiate inflammation and DOMS, some studies reported mechanical hyperalgesia after eccentric exercises without any signs of muscle damage. This supports the crucial roles of NGF and GDNF in DOMS and suggests that the mechanical hyperalgesia development may be associated with inflammation in the extracellular matrix (Peake J. M. et al., 2017). NGF and GDNF are also known to play a role in pathological pain conditions and are increasingly recognized as active players in the whole pain process and upregulated in ischemic skeletal muscle (Turrini et al., 2002). NGF is increasingly regarded as an active player in the whole pain process (McKelvey et al., 2013). Thus, advances in the understanding of the mechanisms and cellular origins of muscle soreness could lead to development of effective interventions for not only exercise-related muscle soreness, but also common myalgia.

## Eccentric Training-Induced Adaptations

### Molecular Aspects

The distinct features of the eccentric contraction compared to other contraction modes are the source of specific training adaptations. A significant body of evidence suggests that compared to concentric contractions, chronically performed eccentric contractions promote greater gains in strength, muscle mass and neural adaptations (Reeves et al., 2009; Roig et al., 2009). The mechanisms responsible for these adaptations are underlined by modifications in gene expression. Indeed the process of exercise-induced adaptations in skeletal muscle involves multiple signaling mechanisms initiating transcription of specific genes that enable subsequent translation into a series of new proteins (Coffey and Hawley, 2007). Several studies have reported that eccentric and concentric actions activate distinct muscular molecular pathways in humans (Kostek et al., 2007) and in rats (Chen et al., 2002). It has been shown that eccentric exercise triggers a progressive activation of genes responsible for cellular growth and development, involved in muscular cell hypertrophy processes. The expression levels of these genes are more stimulated by eccentric actions than by isometric or concentric actions (Chen et al., 2002; Barash et al., 2004; Kostek et al., 2007), presumably due to the unique mechanical stress placed on the eccentrically contracted muscles. For example, in skeletal muscle, the effect of eccentric training was greater than concentric training for liver-type insulin-like growth factor I and mechano-growth factor (positive regulators of muscle growth) (Barash et al., 2004). Such modifications in gene expression profiles are thought to be regulated by mechanical signaling pathways involving proteins that are sensitive to the mechanical status of muscle cell (i.e., Microtubules-Associated Proteins or MAP proteins) (Hentzen et al., 2006). Transcriptome analyses in eccentric-exercised muscles also revealed substantial transcriptional activity related to the presence of leukocytes, immune-related signaling and adaptive remodeling of the intramuscular extracellular matrix until 96 h after exercise (Neubauer et al., 2014). In comparison to concentric or isometric contractions, eccentric contractions appear to upregulate muscle cell activity and anabolic signaling pathway to a greater extent (Douglas et al., 2017).

### Specific Muscle Adaptations to Chronic Eccentric Exercise

**FIGURE 2:**
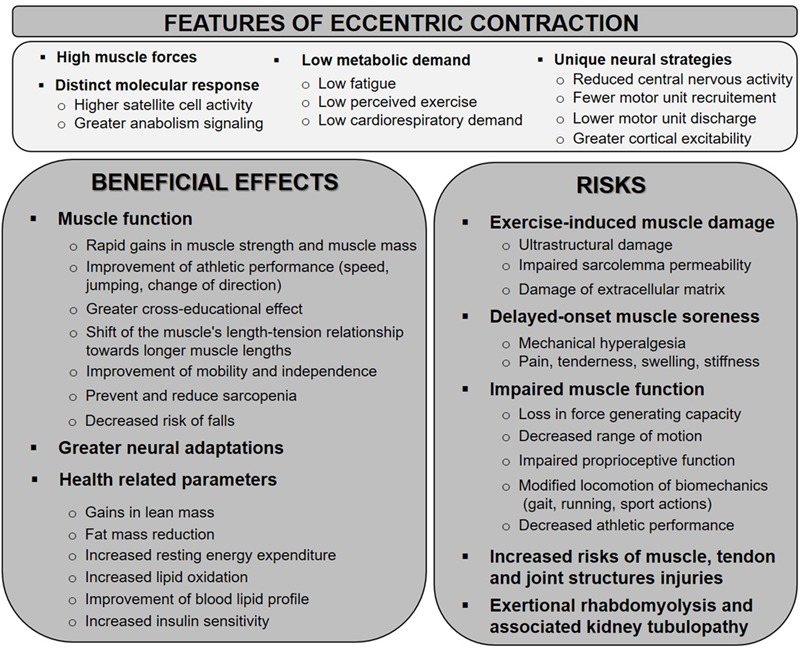
Schematic representation of the potential mechanisms associated with eccentric exercise-induced muscle damage and involved in the development of DOMS. BD, bradykinin; CK, creatine kinase; DOMS, delayed-onset muscle soreness; E–C, excitation–contraction; GDNF, glial cell line-derived neurotrophic factor; IL, interleukin; NGF, nerve growth factor; ROS/RNS, reactive oxygen and nitrogen species; TNF, tumor necrosis factor.

Because the eccentric contraction differs from other contraction types notably in terms of force generation, maximum force produced and energy cost, it could provide different stimuli leading to distinct muscular and functional adaptations (Figure 2) (Franchi et al., 2017a). A significant body of evidence have suggested the superiority of eccentric resistance training in terms of muscular hypertrophy over concentric or conventional strength trainings (Julian et al., 2018). Some studies also reported earlier increments in muscle mass with eccentric-based resistance training when compared with concentric training. However, the findings appear extremely variable to clearly confirm greater gains in muscle mass following eccentric modalities (Julian et al., 2018). Indeed, in their review, Franchi et al. (2014) draw the conclusion that the changes in muscle size are similar between eccentric and concentric training when matched for load or work. A systematic review and meta-analysis about the contribution of the different muscle actions to muscle growth showed a greater muscle mass gain with eccentric contractions but the results did not reach significance (Schoenfeld et al., 2017). Nevertheless, taking into account the energy demand to produce similar force or work, eccentric exercise may be considered as more efficient (Julian et al., 2018). Interestingly, contraction type tends to induce a region-specific hypertrophy. Greater increase in distal muscle size has been observed following eccentric training, while concentric training favors median-muscle hypertrophy (Franchi et al., 2014). In addition, the hypertrophic responses to eccentric versus concentric contractions might be obtained by different structural adaptations mediated by distinct myogenic and molecular responses. While both training regimes appeared to increase muscle fascicle length and pennation angle (Blazevich et al., 2007), conventional strength training would increase pennation angle more than eccentric training. In contrast, eccentric-only resistance training seems to favor fascicle length increase (Reeves et al., 2009), with the implication that eccentric training is able to shift the optimum of the length-tension relationship to longer muscle length (Hoppeler, 2016). This muscle architectural change appears thus particularly interesting for injury prevention and athletic performance (Brughelli and Cronin, 2007). Regarding muscle thickness, similar increases have been observed with both training modes. There are evidence that eccentric training promotes significantly greater increase in muscle strength, whereas the differences in isometric and concentric measures seems less significant (Roig et al., 2009). Findings also showed that the increase in eccentric strength after eccentric training is greater than the gain in concentric strength after concentric training (Vikne et al., 2006). The systematic review of Douglas et al. (2017) also reported mode-specific strength increase and revealed that greater overall strength increases can be achieved after eccentric training than concentric or traditional training. Furthermore, in comparison with concentric exercise, eccentric actions have been reported to induce a greater cross-education effect. Only few studies examined changes in muscle power. Performance in actions involving muscle power or stretch-shortening cycle (such as vertical jump) appeared to be improved to a greater extent with eccentric training compared with concentric or traditional resistance training (Liu et al., 2013; Douglas et al., 2017).

### The Repeated-Bout Effect (RBE)

Skeletal muscle exhibits an intriguing plasticity to repeated bouts of eccentric exercises. Among the adaptations specifically triggered by the eccentric contraction, some contribute to the RBE, aiming thus to protect muscle against EIMD. A large number of theories have been proposed to explain the RBE, suggesting a multifactorial origin of this adaptive process. Potential adaptations have been categorized as (Lindstedt et al., 2001) neural, (Abbott et al., 1952) mechanical and (LaStayo et al., 2003b) cellular theories (McHugh et al., 1999a; McHugh, 2003). However, although many studies have attempted to elucidate the mechanisms behind the RBE, a unified theory is not yet available.

According to the neural theory, the EIMD results from the high mechanical stress imposed on a small number of active muscle fibers during intense eccentric contractions. Although not commonly accepted, this theory also supports a preferential recruitment of fast-twitch motor units during eccentric contractions to explain the higher susceptibility to disruption of the fast muscle fibers. Therefore, it has been postulated that changes in neural activation may contribute to reduce subsequent myofibrillar damage (McHugh, 2003). Suggested neural adaptations involve improved motor units (MUs) synchronization and activation of a large pool of MUs, mainly by recruiting a greater number of slow-twitch fibers (Warren et al., 2000; Chen, 2003; Starbuck and Eston, 2012). Such mechanisms would allow a better distribution of the workload over a greater number of active muscle fibers in repeated bouts (Nosaka and Clarkson, 1995). The fast-setting adaptations but also the existence of contralateral protective effect (Howatson and van Someren, 2007; Starbuck and Eston, 2012; Hody et al., 2013b) support the contribution of neurophysiologic processes in the RBE. Indeed, some studies have reported that an initial bout of eccentric exercise in one limb provides protection from the symptoms of EIMD during a second eccentric bout in the contralateral limb. Nevertheless, the magnitude of protection in the contralateral limb is lower than that observed in the ipsilateral limb, indicating that neural adaptations cannot entirely explain the RBE (Howatson and van Someren, 2007). Moreover, the demonstration of RBE with electrically stimulated eccentric contractions (Black and McCully, 2008) suggests that the RBE can occur independently of neural adaptations and involves thus a peripheral and/or muscular adaptation.

The mechanical origin of initial muscle damage has led authors to suggest that changes in mechanical properties of the musculoskeletal system could render the muscle more resilient to EIMD. With respect to this hypothesis, both the passive and dynamic stiffness of the muscle-tendon complex has been shown to increase after eccentric training (Howell et al., 1993; Reich et al., 2000). These modifications have been, respectively, attributed to an increase in intramuscular connective tissue improving the ability to withstand myofibrillar stress and to a reinforcement of intermediate filament system, in charge of maintaining the alignment and structure of the sarcomeres (i.e., titin, desmin) (McHugh, 2003). In agreement with the reorganization of cytoskeletal proteins, the level of certain structural proteins, such as desmin, was found to increase in the days following eccentric exercise (Feasson et al., 2002; Lehti et al., 2007). This suggests that muscle-specific cytoskeletal remodeling could play a role to protect from future sarcomere disruption. Desmin, the major protein of the muscle intermediate filament, would act as mechanical integrator for the repair of the filaments (Yu et al., 2002). Its reinforcement secondary to transcriptional upregulation may provide mechanical protection from future sarcomere disruption (Peters et al., 2003). However, some studies showing that stiffer muscles are more prone to damage questioned the mechanical theory (McHugh et al., 1999b). Moreover, desmin knockout (KO) mice have been found to exhibit less exercise-induced than wild-type mice (Sam et al., 2000). This finding was, however, imputed to more compliant muscles of KO mice. Recent works have suggested that in addition to their function of structural support to the cell, the intermediate filaments may play an active role in biological processes such as signaling, mechanotransduction and gene regulation. The mechanisms behind these processes are not well understood. Desmin, which is responsible for transmission of stress among myofibrils appears to be required for the maintenance of myofiber alignment, nuclear deformation, stress production and JNK-mediated stress sensing (Palmisano et al., 2015). Growing evidence supports the role of the skeletal muscle intermediate filaments as a stress-transmitting and stress-signaling network. Notably, cytoskeletal proteins help mitochondria not only in their movement and proper cellular positioning, but also to maintain their biogenesis, morphology, function, and regulation of energy fluxes. The functionality of these cytoskeletal proteins may thus influence the mitochondria functions, including the regulation of Ca^2+^ signals and apoptosis (Mado et al., 2019). Remodeling of the intermediate filaments network may also impact cell migratory behaviors important to development (Sanghvi-Shah and Weber, 2017).

Another group of theories explaining the RBE relies on cellular adaptation. Given the focal feature of muscle damage, speculation has been made that the muscle becomes more resistant to EIMD thanks to the removal of stress-susceptible fibers or sarcomeres resulting from the initial eccentric bout (Armstrong, 1984; Newham et al., 1987). However, this is inconsistent with the fact that the initial bout does not have to cause appreciable damage to confer a protective effect. Several works provided evidence that eccentric exercise promotes an increase in series sarcomeres (Lynn and Morgan, 1994; Yu et al., 2004). Such longitudinal addition of sarcomeres is thought to contribute to the protective effect as it would avoid the sarcomere stretching beyond their overlap and thus, their disruption. The sarcolemma and sarcoplasmic reticulum would also become stronger following the initial bout of eccentric exercise (McHugh, 2003). This may limit perturbations of calcium homeostasis and thus, may prevent the calpain activation and the degradation of cytoskeletal proteins. The reduced calpain activity could then explain the attenuation of mitochondrial dysfunction following chronic exposure of eccentric exercise. Other potential adaptations such a decreased susceptibility to calcium-induced mPTP (mitochondrial permeability transition pore) opening or upregulation of heat shock proteins, in particular Hsp70, may also be contribute to protect mitochondrial function (Rattray et al., 2013). In addition, changes in the inflammatory response, such as a reduced activation of the monocytes and neutrophils, have been described after repeated eccentric bouts and may also be related to the RBE (Pizza et al., 1996). Nevertheless, whether adaptation in the inflammatory process is the cause or a consequence of reduced muscle damage is not elucidated. Adaptation may also rely on the monocyte chemoattractant protein 1 (MCP-1), a chemokine involved in activation and attraction of inflammatory cells. Indeed, MCP-1 is dramatically overexpressed at the transcript level after a single bout of eccentric exercise and it appeared even more upregulated after a second bout (Hubal et al., 2008). Authors have thus suggested that MCP-1 enhances muscle recovery after a repeated bout of eccentric exercise via improved signaling between macrophages and satellite cells. Other chemokines may contribute to the protective adaptation to exercise-induced muscle damage. Upregulation of CCL2 and a decreased of NF-kB DNA-binding activity occur following repeated bouts of eccentric exercise. These observations supports the hypothesis that the immune response becomes more efficient to promote the regeneration of muscle tissue after an initial bout of eccentric exercises, notably through enhancement in inflammatory cell infiltration into the muscle and myoblast proliferation (Peake J. et al., 2005). Furthermore, a remodeling of the surrounding extracellular matrix might also occur during the RBE. A strengthening of the extracellular matrix such as an improved integrin support may help to recover faster after eccentric contractions (Hyldahl et al., 2015).

Other potential cellular adaptations include increased protein synthesis, adaptation in the excitation-contraction coupling and increased stress proteins (i.e., heat shock proteins) (McHugh, 2003). In particular, the role of heat shock proteins (HSPs) in protection against muscle damage constitutes an exciting new area of research. The small HSPs (sHSPs) named HSPB1 (Hsp27) and alphaB-crystallin, seem to play important roles in cellular adaptation as they have been implicated in the chaperoning of unfolded proteins, the stabilization of the cytoskeleton as well as in the regulation of the cellular redox state and inhibition of apoptosis (Orejuela et al., 2007). Following one bout of eccentric exercise, the sHSPs translocate from the cytosol to the cytoskeletal/myofibrillar compartment, presumably to stabilize and protect the myofibrillar filament organization (Paulsen et al., 2007, 2009; Frankenberg et al., 2014). Such an observation was not found after concentric exercise (Frankenberg et al., 2014). AlphaB-crystallin interacts with desmin intermediate filaments and, Hsp27, together with alphaB-crystallin, has been suggested to interact with various microfilaments (Orejuela et al., 2007). These data strongly support the idea that alphaB-crystallin and Hsp27 are crucial for the maintenance and the remodeling of myofibrillar structures. Therefore, in line with the reinforcement of the cytoskeletal/myofibrillar structures, appropriate adaptation in the protection systems of HSPs might be important as well (Paulsen et al., 2007). Moreover, because the HSPs are involved in the development of stress tolerance against several stressful insults, it is likely that the HSPs response elicited by an initial damaging bout bestows resistance to a second potentially damaging exercise. Only few studies investigated the HSPs response to repeated bouts of eccentric exercise. Paulsen et al. (2009) revealed that two bouts of maximal eccentric exercise separated by 3 weeks resulted in comparable increased levels of HSPs in the cytoskeletal fraction, despite less damage inflicted during the second bout. The large amount of Hsp27, alphaB-crystallin, and Hsp70 in the cytoskeletal compartment after the repeated bout suggests that a more efficient translocation of these HSPs is plausibly a mechanism behind the RBE. Similarly, Thompson et al. (2002) reported a similar relative increase of Hsp27 and Hsp70 2 days after the first and second eccentric bouts, but, intriguingly, the basal levels of these HSPs appeared to be lower before the second bout. This finding casts doubt on the HSPs as important players in the RBE. Contrary to these findings, results from a study by Vissing et al. (2009) did not point out a role for the HSPs in reducing EIMD, as they observed a blunted translocation of HSPs after the second bout. In this latter study, the low degree of muscle damage inflicted during the exercise and/or the long duration between bouts (8 weeks) could explain the lack of HSPs movement following the second bout. Future studies appear thus to be necessary to delineate the HSPs response after repeated eccentric bouts.

Finally, using a proteomic approach, a short isokinetic eccentric training in human quadriceps was found to induce proteome modifications that suggest an isoform shift in fiber type components (Hody et al., 2011). Indeed, a decreased expression of several glycolytic enzymes coupled with a lower expression of the fast isoforms of some contractile and structural proteins was observed after five sessions of submaximal eccentric contractions. Adaptation in the muscle fiber typology following eccentric training was further supported by a study in mice. This highlighted significant changes in the size and number of muscle fiber types following eccentrically biased trained in comparison with untrained or concentrically biased trained mice: the eccentric training specifically resulted in an increased proportion of slow and fast oxidative muscle fibers (Hody et al., 2013a). Nevertheless, whether a shift to a more oxidative muscle phenotype is really involved in protection against EIMD is still a question to resolve.

## Multiple Applications of Eccentric Training

Eccentric training has sparked a growing interest over the last decade, particularly in light of the emerging health-related benefits of improved muscle mass. Moreover, because a greater volume of exercise can be done at less metabolic and cardiorespiratory cost, eccentric muscle work constitutes a promising training strategy, not only to improve athletes’ performances, but also to help maintain or restore the exercise capacity and quality of life in individuals with reduced tolerance for physical activity (i.e., the elderly or patients with chronic disabilities) (Gault and Willems, 2013; Hyldahl and Hubal, 2014; LaStayo et al., 2014). The lower perceived exertion to perform eccentric exercises helps to increase the adherence of patients to exercise programs. As eccentric contractions have traditionally been associated with muscle damage, the prescription of eccentric training programs in clinical practice has been discouraged for a long time. Nowadays, it is well accepted that when the duration, frequency and intensity of the eccentric training sessions are progressively increased, symptoms of damage can be minimized and even avoided (Croisier et al., 1999; Chen et al., 2013). Additionally, it is now accepted that neither muscle damage nor inflammation are prerequisites for stimulating positive muscle adaptations as protection against EIMD or increased muscle mass (LaStayo et al., 2007). Eccentric training interventions are thus considered as a safe and suitable alternative to traditional resistance exercise. Before discussing the numerous applications of eccentric training, it should be mentioned that the identification of its specific effects in comparison to the other training modalities remain difficult due to methodological reasons. First, the training programs used in several studies involved usual daily movements that do not isolate eccentric and concentric contractions. Secondly, a limited number of studies employed an appropriate calibration of the eccentric and concentric exercises, making conclusive comparison between the contraction modes impossible. Since they imply different metabolic cost for the same mechanical work, eccentric and concentric exercises must be matched for similar mechanical output, metabolic rate or oxygen consumption level, or similar total training load. Additionally, the techniques and equipment to perform eccentric exercises is often sophisticated, require specific experience and may represent financial constraints. These reasons may contribute to the few number of studies comparing eccentric training with other modalities and the difficulty to draw definitive conclusions (Julian et al., 2018).

### Competitive Sports

While few studies have been devoted to the effects of eccentric training in elite athletes compared to untrained subjects, the systematic inclusion of eccentric-based protocols into training programs is recommended for most competitive sports for performance enhancement or injury prevention purposes (Isner-Horobeti et al., 2013; Vogt and Hoppeler, 2014). Indeed, because of its distinct characteristics, eccentric training modalities can further enhance maximal muscular strength and optimize improvements to power, optimal muscle length for strength development, as well as coordination during eccentric tasks (LaStayo et al., 2003a). Eccentric training may also be especially efficient in enhancing speed performance or in rebound activities such as jump (Franchi et al., 2017b; Chaabene et al., 2018). This has notably been demonstrated in basketball players. Those subjected to eccentric training for 6 weeks exhibited a significant improvement in jumping height of 8% while the performance of the players that performed traditional weight-lifting was unchanged (Lindstedt et al., 2002). A change in titin protein isoform has been proposed to explain the increased stiffness of the muscle-tendon unit and enhanced recovery of elastic strain energy (Hoppeler, 2016). These functional adaptations in skeletal muscles are based on increases in muscle mass, fascicle length, number of sarcomeres, and cross-sectional area of type II fibers. In terms of injury prevention, the isokinetic assessment of muscle function, in particular through the eccentric mode, appears to be of great importance for detecting athletes at high risk of injuries before the start of the season (Croisier et al., 2002; Forthomme et al., 2013). Moreover, preventive interventions with controlled eccentric exercises have been shown to decrease the risk of hamstring injury in professional soccer players (Croisier et al., 2008) or of shoulder pain in volleyball players (Forthomme et al., 2013).

### Rehabilitation

Over the last 20 years, eccentric muscle actions have been frequently integrated in the treatment of several pathologies of the locomotor system (Croisier et al., 2009). In particular, chronic eccentric exercise has become a mainstay in the treatment of tendinopathies mainly of the Achilles, patellar and lateral epicondylar tendonitis (Croisier et al., 2007; Hoppeler and Herzog, 2014; Kjaer and Heinemeier, 2014). To justify the relevance of eccentric exercise for strengthening tendon tissues, a stimulating impact of such exercise on collagen synthesis and an increase in blood flow around tendon cells after eccentric actions have been proposed (Guilhem et al., 2010). Eccentric intervention has also been shown to be safe and effective after anterior cruciate ligament reconstruction (ACLR). The studies of Gerber et al. (2007, 2009) reported superior short and long-term results in strength, performance and activity level after surgery when eccentric exercise is part of the rehabilitation after ACL-R in comparison to standard rehabilitation programs. Otherwise, ipsilateral eccentric training has been demonstrated to increase muscles’ strength in the contralateral homologous muscle group, and this in a greater extent than concentric training (Higbie et al., 1996; Hortobagyi et al., 1997). Thus, implementing unilateral eccentric contractions in rehabilitation programs could improve the muscle function of the opposite injured limb without it being solicited.

### Sarcopenia

Given the ever-increasing aging population, the development of strategies to improve the quality of life of the elderly has become a major concern. One of the most evident and disabling consequences of aging is sarcopenia, a process characterized by a progressive and steady loss of lean skeletal muscle mass. Muscle loss is also associated with an increase in intramuscular fat and connective tissue, a reduction in muscle strength, in addition to cardiovascular dysfunction reducing aerobic capacity (Gault and Willems, 2013). Such changes contribute to a decline in functional independence and severely compromise the function, quality of life, and life expectancy in older individuals. Multiple lines of evidence suggest that exercise training can prevent or reverse muscle aging. Indeed, studies comparing muscle characteristics of highly trained young and senior athletes demonstrated that trained subjects can maintain and improve muscle function regardless of their age (Roig et al., 2008; Dickinson et al., 2013). However, the implementation of conventional resistance training programs in the elderly may be hampered by the difficulty of such programs as reduced initial levels of force and cardiovascular dysfunction are frequent in old adults. Conversely, eccentric training programs can massively overload the muscular system with a low cardiopulmonary stress. Interestingly, numerous studies reported that older individuals exhibit a relatively preserved capacity of producing eccentric strength. Indeed, when compared to concentric or isometric strength, the magnitude of the age-related decline in eccentric strength is less pronounced. This provides an additional advantage for eccentric exercises to initiate resistance training and rehabilitation programs (LaStayo et al., 2003b; Roig et al., 2010). In addition to the suitability of eccentric training in old individuals, it is important to emphasize that resistance training with eccentric contractions induces greater beneficial effects than concentric training to improve mobility and independence of the elderly. As in young individuals, high-intensity eccentric resistance training has been shown to be more effective than concentric training in increasing muscle strength and mass in older adults (LaStayo et al., 2003a; Reeves et al., 2009). Other appreciable benefits resulting from eccentric training in old individuals are the improved ability to complete functional tasks and the decreased risk of fall (Gault and Willems, 2013). LaStayo et al. (2003a) demonstrated that using eccentric modality in very frail elderly (mean age, 80 years) was more efficient to reverse sarcopenia and its related functional limitations than traditional weight training. Indeed, the elderly who performed 10–20 min of eccentric resistance exercise 3 times per week over 11 weeks showed significant improvements in strength (60%), balance (7%), stair descent (21%) abilities and a reduced risk of fall. These positive outcomes were not found in the elderly subjects submitted to traditional resistance exercises. Additionally, the subjects of the eccentric group reported the training to be relatively effortless. Besides resistance training, eccentric endurance exercise involving large muscle groups (ECC cycling, downhill treadmill walking, and stepping) seems to be particularly convenient for the elderly (in particular for frail elderly). This training modality minimizes the substantial mechanical stress on single joints occurring during resistance training and provides benefits for strength, muscle mass and potentially aerobic adaptations (Gault and Willems, 2013; LaStayo et al., 2014). The study of Mueller et al. (2009) compared the effects of a moderate load eccentric exercise on an eccentric ergometer to a conventional resistance exercise training. Both trainings were carried out for 12 weeks with 2 sessions per week. A significant increase in maximal isometric strength (8.4%) was observed only for the eccentric group (Mueller et al., 2009). Improvements in body composition characterized by a decrease in intramyocellular lipid content concomitantly with total body fat have also been observed in the elderly after 12 weeks of eccentric ergometer training (Mueller et al., 2011). In contrast, tight lean mass increased similarly after both training modalities. Interestingly, the gain in muscle mass in the elderly following eccentric training was not paralleled by an increase in muscle fiber cross-sectional area (hypertrophy) as observed with traditional exercise training (Mueller et al., 2011). Muscle growth after eccentric training thus seem to occur by the addition of sarcomeres in series or by hyperplasia. While available evidence suggest that eccentric training protocols are well tolerated in elderly individuals, it should kept in mind that old adults show an increased vulnerability to exercise-related muscle damage. Indeed, biopsies from the human *m. vastus lateralis* immediately after a bout of eccentric cycling showed disorganization of sarcomeres, with a higher percentage of disorganization in older (59–63-years) compared to younger adults (20–30-years) (Manfredi et al., 1991). Therefore, careful and safe progression of the intensity of eccentric training is thus strongly advised when initiating eccentric programs in the elderly.

### Chronic Diseases

Musculoskeletal dysfunction is relatively common in patients with chronic conditions such as chronic obstructive pulmonary disease, chronic heart failure or stroke (Hyldahl and Hubal, 2014). Although the exact etiology of the muscle function decline in these patients is not yet clear, it is believed that the lack of physical activity contributes at least to some of the deleterious changes in muscle function (Roig et al., 2008). Moreover, the ability of exercise to maintain mobility and minimize muscle wasting in most people with chronic conditions is commonly accepted. Until now, only few studies explored the use of eccentric-biased programs in persons with chronic health conditions. Nevertheless, current evidence exists regarding the effectiveness and safety of eccentric exercise in restoring musculoskeletal function in patients with different chronic conditions. For instance, compared to conventional training programs, judicious eccentric-based protocols result in greater strength gains and enhancement of functional capacity in cancer survivors, Parkinson disease patients or total knee replacement patients (Hyldahl and Hubal, 2014). However, such favorable effects were not observed in individuals with multiple sclerosis (Hayes et al., 2011). Studies exploring the use of resistance training in individuals recovering from a stroke revealed that eccentric contractions were more effective for improving neuromuscular activation, strength, and walking speed than concentric contractions (Engardt et al., 1995; Clark and Patten, 2013). Since eccentric training seems to provide greater central neural adaptation than concentric modes of exercise, the use of eccentric exercise may be particularly effective for patients with central nervous system diseases. The physiologic characteristics of eccentric contraction (attenuated cardiopulmonary stress, low metabolic cost) seem to be well suited for their incorporation into the revalidation of patients intolerant to intense cardiac and respiratory efforts (i.e., patients with heart disorders or lung pathologies) (Meyer et al., 2003; Roig et al., 2008). Eccentric training has been suggested to attenuate reductions in arterial compliance, thus potentially limiting the risks commonly associated with resistance training in patients with coronary disease (Okamoto et al., 2006). Steiner et al. (2004) compared concentric and eccentric training at similar heart rate (85% of HR) in patients suffering from cardiac problems. Training was carried out 3 times per week during 8 weeks, with a progressive increase of the exercise intensity the first 5 weeks. The authors showed a significant gain in muscle torque following the eccentric training. Both training modalities induce a small 3% increase in leg muscle mass but leg and whole body fat mass appeared to decrease only in patients trained eccentrically. Interestingly, despite working at fourfold higher mechanical loads, the eccentric group did not show different changes in cardiovascular variables (such as heart rate, mean arterial pressure, or vascular resistance) than the concentrically trained subjects (Meyer et al., 2003). Collectively, all studies reported eccentric exercise to be a safe training modality for patients with various cardiac conditions.

Eccentric exercise may also be useful in the prevention or treatment of metabolic diseases given its rapid and favorable effects on health related parameters (Roig et al., 2008; Paschalis et al., 2010; Isner-Horobeti et al., 2013). For instance, eccentric training is more effective to improve glucose tolerance than concentric training. Additionally, Paschalis et al. (2010) demonstrated that a weekly bout of intense eccentric exercise – and not concentric exercise – is sufficient to improve health risk factors. They found that only 30 min of eccentric exercise per week for 8 weeks markedly increased resting energy expenditure and lipid oxidation as well as decreased insulin resistance and blood lipid profile. The study of Marcus et al. (2008) compared the effects of a 16-week aerobic exercise training alone to aerobic exercise combined with moderate load eccentric exercise in diabetes type 2 patients. While glycemic control and physical performance were similarly improved in all patients, the improvements in tight lean mass and body mass index were larger when eccentric exercise was performed.

In regard with muscular dystrophy pathology, no human study investigated the potential effects of eccentric training in this disease. It is likely that eccentric contractions may accelerate the degenerative process given that the degenerative nature of dystrophic muscle can partially be accounted for by exhaustive regenerative cycles (Hyldahl and Hubal, 2014). Nevertheless, some recent animal studies indicate a favorable adaptation to moderate exercise in dystrophic animals (Lovering and Brooks, 2014). In fact, despite the increased vulnerability of dystrophic (*mdx*) muscles to eccentric contractions, young *mdx* mice were found to recover from and adapt more quickly to EIMD than wild-type mice (Ridgley et al., 2009; Call et al., 2011). However, such increased regenerative capacity was lost in older animals (Carter et al., 2002) and it is still unclear whether an eccentric-based training program would be helpful or detrimental to the long-term health of the muscle.

Notwithstanding recent evidence demonstrate the benefits of eccentric training interventions in several fields, there is a real need to further study the physiology of eccentric contraction. Indeed, it is still unclear whether this high specificity of eccentric training adaptations compromises the transferability of strength gains to more functional movements (Roig et al., 2010). Moreover, long-term implications of eccentric training in old individuals or in patients with chronic diseases should be explored in more details. Likewise, further investigations are required to optimize parameters as intensity, duration, and modes of eccentric training leading to the favorable effects on muscle performance, health and quality of life.

## Practical Considerations

Eccentric actions can be integrated in different types of muscle training. Plyometric exercises, such as drop jump, is frequently used to improve speed and jumping ability in athletes. The literature recommends specific habituation training and knowledgeable supervision due to the inherent risk of injuries in such exercises (Hoppeler, 2016). Eccentric based resistance training, characterized by high muscle loads at low metabolic cost, has been increasingly prescribed for individuals with a centrally limited exercise tolerance (LaStayo et al., 2014). However, in most patient populations, the use of high mechanical loads may constrain their adherence to resistance muscle training. Therefore, the new modality “moderate load eccentric exercise” represents an attractive choice in various medical conditions (Hoppeler, 2016). Over the last decades, various motorized ergometers or similar devices allowing safe and controlled application of eccentric loads, have been developed for rehabilitation and performance purposes.

The prescription of eccentric muscle training require specific experience. Practitioners must respect fundamental precepts and consider important safety considerations concerning the applications of eccentric muscle training, especially during the initial implementation phase. Exercise professionals should be aware of the potential detrimental effects of eccentric contractions as well as the ways to prevent their occurrence. Nowadays, it is well accepted that repeated exposure to eccentric exercises confers protective adaptations against potential further damage (McHugh, 2003; Nosaka and Aoki, 2011). Even if the magnitude of this “repeated-bout effect” appeared larger if the initial eccentric bout involved high workloads, this strategy could be problematic, especially for those undergoing rehabilitation or elite athletes. Indeed, DOMS and the functional consequences associated with EIMD may frequently disturb the progress of rehabilitation and/or training programs. Moreover, the uncomfortable sensations may discourage people to continue exercise training. Therefore, an initial phase consisting of submaximal eccentric muscle actions with incremental loading over multiple sessions should be used to introduce individuals to eccentric muscle training (LaStayo et al., 2003b). Flann et al. (2011) demonstrated that a 3-week gradual “ramp up” eccentric protocol was effective at promoting muscle hypertrophy in the absence of demonstrable markers of muscle damage. In clinical interventions, the progressive ramping eccentric protocol typically starts with load of 50–75 W to reach the target training load of 400–500 W. Higher loads, over 1,200 W, can be achieved in competitive athletes (Hoppeler, 2016). A period of 2–4 days between the exposure stimulus and progressively higher levels of loading has been suggested as optimal (Hoppeler, 2016). Guidelines to design ramping protocols in rehabilitation conditions are described in more details by LaStayo et al. (2014). The exercise duration generally increases from 5–10 min to 20–30 min over the sessions. When using higher load trainings, four bouts of 5 min seems to be equally effective and less tiring for subjects (Steiner et al., 2004; Vogt and Hoppeler, 2014). A training frequency of two sessions per week seems to be the lower limit to induce measurable gains (Mueller et al., 2009).

When conceiving eccentric interventions, practitioners should also take into account the parameters affecting the extent and duration of EIMD and/or slower recovery. Exercises performed at high vs. low eccentric torque, at long vs. short muscle length and increasing numbers of eccentric contractions appear to result in more severe EIMD (Nosaka and Newton, 2002). Skeletal muscles do not display the same vulnerability to EIMD. The upper limb muscles appear mostly more affected than lower limb muscles and the knee flexors more than the knee extensors (Chen et al., 2011). The untrained status, genetic variations, aging and chronic diseases are other factors increasing the severity of potential EIMD (Tidball, 2011; Gault and Willems, 2013; Baumert et al., 2016). Special attention should thus be given to these populations when establishing the initial eccentric exercise prescription in rehabilitation settings.

Even if trained athletes are generally less affected by EIMD than untrained people when submitting to the same eccentric protocol, they might not escape to the detrimental effects of eccentric contractions in some circumstances. They might be particularly vulnerable to EIMD at the start of the season, or when they return to competition after injury or following an unaccustomed eccentric session (Cheung et al., 2003). The occurrence of EIMD associated with unaccustomed eccentric exercise could be problematic in athletes because of the associated negative consequences on locomotor biomechanics and sport performance within the short term (Cheung et al., 2003; Assumpcao Cde et al., 2013). Moreover, when athletes suffered from DOMS, they are frequently unable to train at their maximal intensity which can compromise the quality of the training programs. Even if the negative functional consequences of EIMD are transitory, it seems important to avoid their onset even in healthy athletes. If EIMD have not been avoided, it is recommended not to perform high intensity exercises, particularly explosive efforts. Indeed, the risk of injuries such as muscle tears or ligament rupture has been shown to increase due to the disturbed muscle function and mechanical fragility. Accordingly, it should be kept in mind that even when muscle hyperalgesia is resolved, a decrease in muscle function may persist. Care must thus be exerted in the days following an episode of DOMS (Damas et al., 2016). When experiencing EIMD, stretching should also be avoided since it could interfere with recovery. Since EIMD triggers inflammation response, some practitioners have used non-steroid anti-inflammatory drugs (NSAIDs) in an attempt to attenuate the clinical symptoms (Paulsen et al., 2012). Nevertheless, studies demonstrated that reducing or blocking potential inflammation response may negatively perturb the muscle cell activity and hinder the hypertrophy and regenerative processes (Mackey et al., 2007). Therefore, NSAIDs should be avoided in healthy subjects (Paulsen et al., 2012). Contrary to this, evidence suggests that NSAIDs may be beneficial for subjects characterized by a low-grade systemic inflammation contributing to sarcopenia (Bautmans et al., 2005; Rieu et al., 2009). In the elderly or in individuals with chronic disease, the use of NSAIDs may help to maintain muscle mass (Rieu et al., 2009).

## Conclusion

The study of eccentric contraction is no longer confined to muscle physiology and sport sciences but is becoming central in clinical medicine and is likely to expand in the near future. Indeed, due to its unique neural, mechanical and metabolic properties, the eccentric mode has gained a growing interest in several fields. In addition to its efficiency in sports performance and rehabilitation, the eccentric training interventions constitute an attractive strategy to prevent muscle wasting in sarcopenia or in many chronic diseases. Increasing evidence also support the beneficial effects of eccentric exercises on body composition and other health-related parameters, making this contraction mode a promising tool for various patient populations. However, unaccustomed eccentric exercise is well known to induce muscle damage that manifests by a range of clinical symptoms including DOMS and decreased muscle function. Up to now, there is no equivocal therapeutic approach allowing a significant attenuation in the symptoms of damage. Conversely, it has been clearly demonstrated that repeated exposures to eccentric actions with progressively increasing loads can prevent the occurrence of muscle damage or DOMS.

## Future Perspectives

Although the eccentric contraction has received more attention over the last decade, many questions remain unanswered with regard to both the initial damaging response to unaccustomed eccentric contraction and the subsequent adaptations. Furthermore, the mechanisms behind the protective effect conferred by a repeated eccentric bout are still in great part speculative. Yet, the numerous practical applications of eccentric exercise in sports, rehabilitation and pathological conditions justify the need to elucidate the mechanisms underlying the acute and chronic effect of eccentric exercise on the skeletal muscle. In addition, a better knowledge of the transient eccentric induced damage and subsequent adaptations on a mechanistic level may help to further understand the degeneration/regeneration cycles in healthy skeletal muscle and to identify abnormalities in these processes in pathological conditions as in neuromuscular diseases. Given some similarities in the histopathological alterations that follow unaccustomed eccentric actions with those observed in muscular dystrophy pathology, further investigations on the eccentric exercise may unravel crucial issues in molecular mechanisms frequently involved in neuromuscular diseases. Investigations employing rigorous standardization of the experimental conditions in the eccentric and other training groups are necessary to determine the specific multi-target and to draw guidelines for eccentric activity prescriptions. In particular, more efforts should be devoted to develop intensity, duration and modes of eccentric training optimizing efficiency of this method.

## Author Contributions

SH wrote the manuscript. J-LC, TB, BR, and PL revised the manuscript according to their respective field of expertise.

## Conflict of Interest Statement

The authors declare that the research was conducted in the absence of any commercial or financial relationships that could be construed as a potential conflict of interest.
